# Editorial to the Special Issue: Food Consumption Determinants and Barriers for Healthy Eating

**DOI:** 10.3390/nu17152403

**Published:** 2025-07-23

**Authors:** João P. M. Lima, Samuel Durán-Agüero

**Affiliations:** 1 H&TRC—Health and Technology Research Center, Coimbra Health School, Polytechnic University of Coimbra, 3046-854 Coimbra, Portugal; 2 GreenUPorto—Sustainable Agrifood Production Research Centre, 4169-007 Porto, Portugal; 3 SUScita—Research Group on Sustainability, Cities and Urban Intelligence, 3030-199 Coimbra, Portugal; 4 Facultad de Ciencias de la Rehabilitación y Calidad de Vida, Universidad San Sebastián, Sede Los Leones, Lota 2465, Providencia, Santiago 7500000, Chile; samuel.duran@uss.cl

The global food environment is undergoing rapid transformation, shaped by economic, social, cultural, and psychological factors that influence dietary behaviors [1,2]. Understanding the determinants and barriers to healthy eating is crucial for developing effective policies, interventions, and strategies to promote public health [3,4,5]. This Special Issue entitled “Food Consumption Determinants and Barriers for Healthy Eating”—aims to advance knowledge in this domain by bringing together cutting-edge research on factors that facilitate or hinder healthy dietary patterns.

Food choices are influenced by a complex interplay of individual, environmental, and systemic factors [1,6]. Individual-level determinants include nutritional knowledge, personal preferences, psychological factors, and sociodemographic characteristics such as age, gender, and income [7]. Environmental influences encompass food availability, accessibility, affordability, marketing strategies, and cultural norms [8,9]. Additionally, systemic barriers such as food insecurity, economic disparities, and policy frameworks significantly impact dietary behaviors.

This Special Issue highlights interdisciplinary research addressing key themes such as

- Psychological and behavioral determinants of food choice and healthy eating habits [10,11];
- Socioeconomic and cultural influences on dietary patterns [12,13];
- The role of food environments and policies in shaping consumption behaviors [14];
- Barriers related to affordability, accessibility, and food literacy [15];
- Innovative interventions and strategies to promote healthier food consumption [11].

The articles featured in this Special Issue contribute valuable insights for researchers, policymakers, and public health practitioners seeking to enhance nutritional well-being at individual and population levels. By understanding the intricate factors affecting food consumption, we can move toward more inclusive and effective strategies to overcome barriers and foster a global culture of healthy eating.

We extend our gratitude to all contributors for their rigorous research and thoughtful discussions. We hope that this collection serves as a valuable resource for future studies and inspires actionable change in the field of nutrition and public health.
